# An Overview of the Recent Developments in Metal Matrix Nanocomposites Reinforced by Graphene

**DOI:** 10.3390/ma12172823

**Published:** 2019-09-02

**Authors:** Mehran Dadkhah, Abdollah Saboori, Paolo Fino

**Affiliations:** Department of Applied Science and Technology, Politecnico di Torino, Corso Duca Degli Abruzzi 24, 10129 Torino, Italy

**Keywords:** metal matrix composite, graphene, powder metallurgy, mechanical properties, thermal properties

## Abstract

Two-dimensional graphene plateletes with unique mechanical, electrical and thermo-physical properties could attract more attention for their employed as reinforcements in the production of new metal matrix nanocomposites (MMNCs), due to superior characteristics, such as being lightweight, high strength and high performance. Over the last years, due to the rapid advances of nanotechnology, increasing demand for the development of advanced MMNCs for various applications, such as structural engineering and functional device applications, has been generated. The purpose of this work is to review recent research into the development in the powder-based production, property characterization and application of magnesium, aluminum, copper, nickel, titanium and iron matrix nanocomposites reinforced with graphene. These include a comparison between the properties of graphene and another well-known carbonaceous reinforcement (carbon nanotube), following by powder-based processing strategies of MMNCs above, their mechanical and tribological properties and their electrical and thermal conductivities. The effects of graphene distribution in the metal matrices and the types of interfacial bonding are also discussed. Fundamentals and the structure–property relationship of such novel nanocomposites have also been discussed and reported.

## 1. Introduction

Composite materials contain two or more distinct constituents which are engineered or naturally occurring, with remarkably various properties (chemical, physical and mechanical) [1,2,3,4,5]. In general, typical metal matrix composites (MMCs) reinforced by ceramic particles or fibers show superior features with respect to the unreinforced alloys [6,7,8,9,10]. In fact, MMCs have the best characteristics of both matrix and reinforcement together; for instance, a MMC can demonstrate the ductile and tough behavior of a matrix, along with the high elastic modulus of reinforcing particles [11,12,13]. Therefore, MMCs have been utilized in various applications, such as in the aeronautics, transportation, marine, and defense sectors [14,15]. In comparison with different reinforcements, like ceramics, carbon fibres (CFs) and graphite particles are found to be desirable reinforcements, because of unique properties, such as simultaneously high thermophysical properties and low thermal expansion coefficients (CTE) [11,16]. The self-lubricating behaviour of MMCs reinforced by graphite or CFs is due to the role of graphite as a solid lubricant. These reinforcements impart MMCs with low coefficients of friction and high wear resistance properties. It should be mentioned that in an atmosphere with specific requirements (like a vacuum, ionizing radiation and plasma) MMCs are particularly suitable [8,9,16,17]. In metallic alloys, CFs and graphite reinforcements impart considerable stability in all dimensions and also prevent mechanical vibrations [12,17,18].

Moreover, it has been found that MMCs reinforced by particles give competitive economical and isotropic characteristics, while those reinforced by continuous fibre offer the highest strength. Given that fact, to strengthen the metallic matrices, CFs can be replaced by inexpensive graphite particulates, especially in the case of MMCs developed for sliding bearing and electrical brush applications [15,18]. To obtain desirable mechanical and self-lubricating properties, such MMCs are conventionally strengthened with micron-size graphite particulates [11,19,20]. For these purposes, the size of graphite particulates is several to hundred micrometres, even up to a few millimetres [18,21,22]. In general, mechanical properties of the resulting composites, like strength, fracture-resistance, self-lubrication and ductility are strongly affected by the size of reinforcing materials. Indeed, by increasing the reinforcement size, the tensile properties of MMCs decreases. MMCs reinforced by large particles are sensitive to crack formation during the tensile testing and consequently result in brittle failure of the composites. As mentioned earlier, by increasing the particle size of ceramic-reinforced composites, all the tensile properties of composites are deteriorated. Thus, it is believed that it is possible to have superior features by decreasing the dimensions of reinforcing materials and/or the grain size of the metallic matrix from the micron size to nano-size, which results in “nanocomposites.” For instance, Zhu et al. reported that by using a carbonaceous nanomaterial, like carbon nanotubes, it would be possible to improve both the mechanical and wear properties of AZ31 alloy [23].

Furthermore, by decreasing the size of reinforcement (to nano-size), it is possible to improve some of the weak properties of metal matrix nanocomposites (MMNCs), like poor ductility, elongation, etc. [11,23,24,25,26,27]. Over the last decade, different types of nanocrystalline have been synthesized by the fast development of nanotechnology [28,29]. In fact, several studies have shown that through the integration of nanotechnology and materials science and engineering, it would be possible to develop new MMNCs with superior properties [30,31,32]. 

In recent years, several studies have been focused on carbonaceous nanomaterials, like graphene and carbon nanotubes (CNTs), as an important category of novel materials for various applications, such as structural and functional ones. This increasing interest in their applications is mainly because of their simultaneous exceptional mechanical properties and excellent electrical and thermal conductivities [33]. Therefore, incorporation of graphene and CNTs in metal matrices results in the development of new MMNCs with improved mechanical, electrical and thermal features. Owing to their high aspect ratio feature (i.e., length to diameter, or length to thickness ratio), carbon nanotubes and graphene have attracted an increasing interest to be used as the most effective reinforcement for production of the new MMNCs [30,34]. According to the published papers, there are various production processes which have been developed for the syntheses of MMNCs reinforced by graphene. As reported, the main processing techniques applied to the fabrication of these MMNCs can be classified into powder metallurgy, melting, solidification and electrochemical deposition. However, has been found that the fabrication of MMNCs reinforced by graphene is faced with several challenges, like the distribution of graphene in the metallic matrices, agglomeration of graphene, formation of a poor interfacial bonding between some of metallic matrices and graphene, and the preferred orientation of graphene in some production processes. Therefore, to achieve an improvement in the final properties, all those challenges should be considered and addressed [35,36]. Table 1 summarises an overview of MMNCs reinforced with graphene as a reinforcement.

There are some motivations to develop MMNCs reinforced by graphene. It was found that several factors from the inherent characteristics of graphene pertaining to its dispersion and the interface of matrix/graphene play key roles in the strengthening of MMNCs reinforced by graphene. Indeed, the introduction of graphene into a metallic matrix and synthesizing a homogeneous, dispersed structure with common production processes remain open to researchers as a challenge. Those challenges are mainly due to the significant difference in the density of graphene and metallic matrices, larger interfacial bonding than carbon nanotubes and a reaction at the interface of matrix and reinforcement due to the reactivity of some metallic materials [67,68,69]. At the same time, a weak interface between the carbonaceous reinforcement and matrix is another significant processing issue. For instance, Wejrzanowski et al. studied the thermal properties of some metallic matrices, like Cu and Ag reinforced by single-layered graphene and multi-layered graphene. They found that for both cases the interface is a barrier for heat transfer with a thermal conductance of at least two orders of magnitude lower than the one of metal itself. Moreover, it was detected that by increasing the number of graphene layers, the thermal boundary conductance decreases. Despite all those challenges in the fabrication of MMNCs reinforced by carbonaceous nanomaterials, there is a growing body of literature that focused on finding solutions for the abovementioned challenges and developing new MMNCs reinforced by graphene with superior properties. Therefore, the aim of this work is to provide a comprehensive overview of the fabrication methods, both conventional and advanced techniques, and a characterization of the main MMNCs reinforced by graphene over the last years. At the beginning, thermal and mechanical properties of carbonaceous nanomaterials, like carbon nanotubes and graphene, are described and compared. Thereafter, various techniques of the most prominent approaches to achieve MMNCs reinforced by graphene, such as spark plasma sintering (SPS) and additive manufacturing (AM) are explained in detail. At the end, all the latest publications in this field are discussed and reviewed. Indeed, the target of this work, contrary to the other available review articles, is not to assemble all the existing literature, but to clarify the importance and opportunities of these innovative materials.

## 2. Carbonaceous Nanomaterials

**Figure 1:**
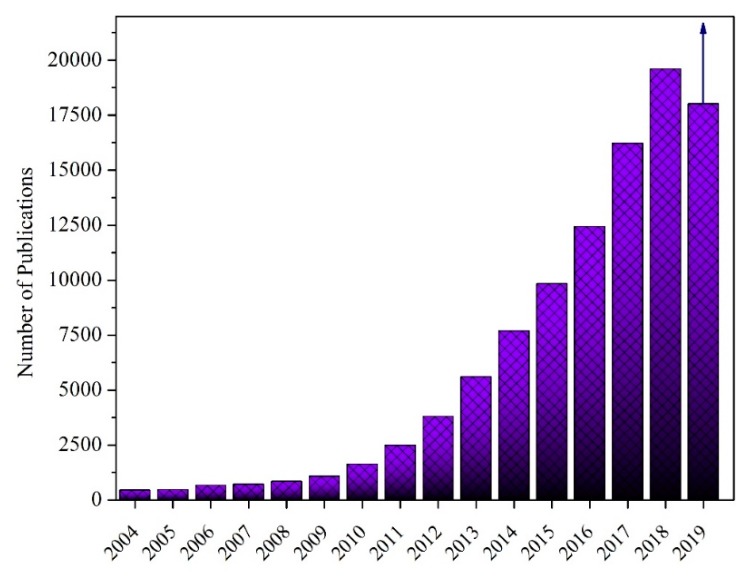
Number of publications in web of science database with the keyword of graphene as a function of year.

Graphene is a single atomic layer of sp^2^- hybridized carbon atoms which has a high packing density in a honeycomb lattice. Graphene has been attracting worldwide attention due to some of its unique properties. It is known as the strongest material measured so far, the most stretchable crystal, the stiffest material and the most thermally conductive material [70,71]. Graphite is the most well-known type of carbon, consisting of many sheets of graphene stuck together on the c-axis. In this case, the interlayer spacing is 0.34 nm and produce strong bonding between carbon atoms, while there is poor van der Waals bonding between the layers. From the thermodynamic point of view, reduction of layer numbers through the exfoliation of graphite was not to be possible [72]. Researchers speculated about synthesizing of graphene during previous decades. In 2004, graphene was productively produced in the lab by Geim et al. at the University of Manchester (UK) through the mechanical exfoliation of graphite crystals employing the cohesive tape method [73]. However, it was found that this method cannot be considered as a proper way for large-scale production. Carrying that in mind, to date, several researchers aimed for the development of different approaches for the mass production of graphene; for instance, the thermal evaporation of silicon carbide [74,75], chemical vapor deposition (CVD), of graphene on metal carbides or metal surfaces [76,77], and wet chemical synthesis of graphene oxides followed by reduction [78,79]. Therefore, the aims of several researchers are the control of graphene films on substrates, functionalizing of graphene as reinforcement and finding the new applications of graphene, as shown in Figure 1 [80,81].

As can be observed in Figure 1, since 2004, extensive studies have been undertaken on graphene and graphene nanocomposites. According to the web of science database, the number of publications on graphene has significantly raised to many thousands of papers (more than 190,000). Therefore, the number of researchers who have focused on the development of new methods to produce graphene has increased significantly.

Generally speaking, graphene can be synthesized by two various methods, including top-down and bottom-up methods. The bottom-up technique consists of the unzipping of carbon nanotubes, epitaxial growth technique [82] and chemical vapour deposition [83], whereas the top-down approach is graphite exfoliation.

Nevertheless, according to the literature, so far, the development of the dispersion graphene strengthened MMNCs with no damage in the intrinsic structures is the most significant challenge. The most essential characteristics of graphene are listed in Table 2.

**Figure 2:**
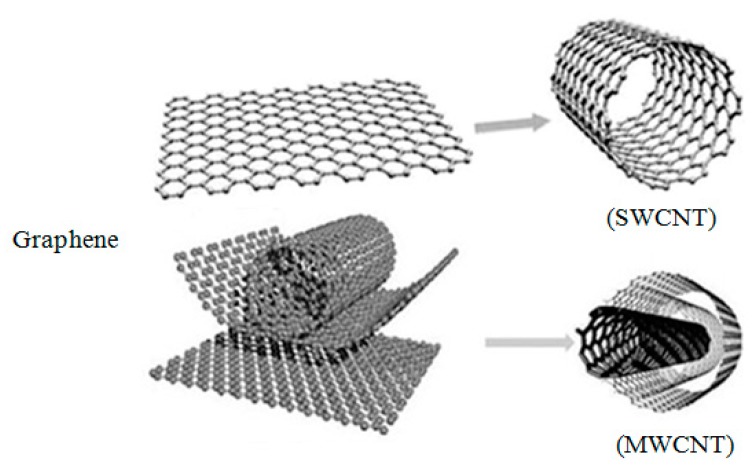
Graphene, single walled carbon nanotubes (SWCNT) and multiwall carbon nanotubes (MWCNT) structures [88].

The story of MMNCs reinforced by carbonaceous nanomaterial started with MMNCS reinforced by carbon nanotube. Indeed, there is a growing body of literature that has studied carbon nanotubes since their discovery in 1991 by Iijima [86]. Carbon nanotubes can be categorized into single-wall (SWNT), double-wall (DWNT) and multi-wall (MWNT). The SWNT forms when a sheet of carbon atoms is wrapped into a seamless cylinder with a diameter of 1–2 nm. While double-wall and multi-wall nanotubes consist of two or more concentric single-walls with diameters of 4 to 20 nm, as shown in Figure 2.

CNTs have unique characteristics—mechanical and physical characteristics that make them very interesting materials. Therefore, they would be a promising candidate for the reinforcing phase in MMNCs, offering several advantages, such as inherent stability at high temperatures, superior thermal and electrical conductivity coming from the metallic matrices and enhanced performance of MMNCs in industrials parts. Nonetheless, it is very challenging to incorporate CNTs into metallic matrices due to their tendency to form agglomerates within the metallic matrices. In addition, owing to a significant discrepancy in the surface tensions of carbon nanotubes and metals, the wetting of carbon nanotubes by molten metals is poor, and accordingly, results in the formation of a poor interfacial bonding [87].

Despite the superior mechanical features of SWNTs, they are not yet extensively employed as reinforcement due to their expensive production and purification methods. However, MWNTs are composed of some centred layers, and they are easier to be produced [89,90].

In recent years, with the progress of nanotechnology, the cost of MWNTs, in particular, those of an industrial grade, has been noticeably decreased. Accordingly, to manufacture the MMNCs with favorable physical and mechanical features, carbonaceous nanofillers with affordable prices, are essential reinforcements. The characteristics of carbonaceous reinforcements are compared in Table 3. As can be seen in this table, graphene simultaneously has the highest thermal conductivity and Young’s modulus, together with the lowest coefficient of thermal expansion with respect to the other carbonaceous nano-reinforcements. Hence, graphene is considered the most important carbonaceous nanomaterial to be used in the development of new MMNCs, with superior properties. Thus, this paper focused on the developments of MMNCs reinforced by graphene.

## 3. Mechanical Properties of Carbonaceous Nanomaterials

According to the latest findings in the literature, to forecast and evaluate the mechanical performance of graphene, both experimental approaches and theoretical simulations have been used; e.g., numerical simulation tools, such as equivalent-continuum, quantum mechanical and atomistic modelling [96]. The Young’s modulus (E) of a single-layered graphite sheet is predicted by Sakhaee Pour around 1 TPa through utilizing the equivalent-continuum concept [97]. Molecular dynamics (MD) are usually employed to model the motion of atoms numerically according to their relevant potential energies. During simulations, for a while, atoms are allowed to interact, which leads to increasing motion and force fields among them. For these purposes, different interatomic potential models, such as the Lennard–Jones, Morse and Tersoff–Brenner models, have been proposed [98]. MD simulations were employed for mechanical performance prediction of single layer of graphite by Tsai and Tu [99], and as a result, an elastic modulus of 0.912 TPa was achieved. Zheng et al. [100] have reported the Young’s modulus of graphene in the forms of armchair and zigzag being equal to 1.086 and 1.05 TPa, respectively.

Experimentally, Lee et al. have determined the Young’s modulus (1.02 TPa) and intrinsic tensile strength (σ, 130 GPa) of single-layer graphene by employing the nanoindentation of the atomic force microscope (AFM) [101]. Furthermore, by applying a similar method, they reported the values of E and σ for a graphene bilayer and trilayer: 1.04 TPa, 126 GPa and 0.98 TPa, 101 GPa, respectively [102]. It should be mentioned that epoxide and hydroxyl groups in GO significantly deteriorate the Young’s modulus of GO. In recent years, large GO sheets with an average area of 272.2 mm^2^ were prepared by Lin et al. through the centrifugation technique, and achieved the best elastic modulus, of ~13.5 GPa [103].

A list of practical Young’s modulus and tensile strength of different carbonaceous nanomaterials is shown in Table 4.

## 4. Thermal Properties of Carbonaceous Nanomaterials

In carbonaceous nanomaterials, thermal energy is transported by phonons’ conduction. The thermal conductivity (k) of single-layer graphene (SLG) was measured by Baladin et al. through confocal micro-Raman spectroscopic measurements. They reported extremely high thermal conductivity (4840–5300 W·m^−1^·K^−1^) for SLG, which was prepared by mechanical cleavage at ambient temperature [113,114]. With respect to the high thermal conductivity of graphene, it was considered a proper material for use in electronic packaging industries. Ruoff et al. obtained the k value of SLG coated on an amorphous silica substrate equal to 600 W·m^−1^·K^−1^ at ambient temperature [115]. That decrease in the thermal conductivity was due to the increase of scattering points as a consequence of defects and the inclusions increment.

In nanotubes, the conduction of a phonon is influenced by some important parameters, including the number of active phonon modes, the average length of the mean free path for the phonons and inelastic umklapp scattering. For this reason, the presence of impurities, and the geometry of the tubes, influence the thermal conductivity of nanotubes [116]. Despite these factors, a fabulous high thermal conductivity of 6600 W·m^−1^·K^−1^ was predicted for SWNTs by theoretical MD simulations at room temperature [117]. Some practical outcomes have been presented in the previous works for the thermal conductivity values of CNTs. Pop et al. measured the thermal conductivity of a suspended SWNT through electrical analysis at 300–800 K to be about 3500 W·m^−1^·K^−1^ [118]. In other research, Honet et al. found the thermal conductivity of 1750–5800 W·m^−1^·K^−1^ for the SWNT ropes at a room temperature by the conventional thermocouple method [119]. As reported by Fuji et al. thermal conductivity of a single MWNT decreases at ambient temperature with increasing tube diameter, based on a suspended sample-attached T-type nano-sensor experiment [120]. For instance, the thermal conductivities of MWNTs with 9.8 and 28.2 nm diameters are 2069 and 500 W·m^−1^·K^−1^, respectively. As reported by Kim et al., the k value of MWNT is more than 3000 W·m^−1^·K^−1^ at room temperature via suspended microdevice measurements [121]. Experimental mechanical and thermal characteristics of traditional CFs and carbonaceous nanomaterials are summarized in Table 5.

## 5. Fabrication of MMNCs Reinforced by Graphene

Over the last decade, several technologies, such as casting, additive manufacturing (AM), thermomechanical processing and powder metallurgy (PM) have been employed to produce metallic monolithic alloys and/or composite materials [122,123,124,125]. Among them, PM is a suitable technique for producing MMNCs with carbonaceous nanofiller owning to its compliance and simplicity. The basic route in this process is a mechanical blending of powders in a rotary mill, so that all materials remain in the solid state, followed by compacting, sintering, cold isostatic pressing (CIP), hot pressing/hot isostatic pressing (HIP) or spark plasma sintering (SPS) to obtain the minimum porosities and the highest density. The main limitation of PM techniques is related to the price of raw material powders, which are expensive [125]. However, it should be noticed that the AM techniques, such as laser powder bed fusion (LPBF) and directed energy deposition (DED) processes, that are layer-wise manufacturing processes, are rapidly growing in the fabrication of MMNCs [126,127,128,129].

As expected, obtaining a homogeneous dispersion of filler by simply mixing carbonaceous nanomaterials with metal powders is entirely poor. However, it is possible to get a better dispersion of reinforcement particles by using high energy milling methods, such as ball milling and mechanical alloying. Since a long-duration ball milling process can damage the crystallinity of carbonaceous materials, the processing steps should be carefully carried out to avoid introducing any defects in these materials [130]. Morsi et al. showed that, by increasing ball milling time, despite a high level of the structural defects, a homogeneous distribution of MWNTs in the Al matrix could be achieved [131]. Poirier et al. reported that milling MWNTs alone leads to the defect formation, but retains the tubular structure [132].

**Figure 3:**
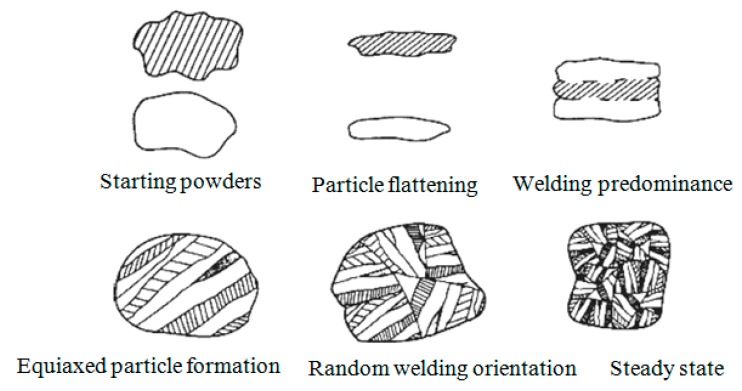
Evolution of various steps of mechanical alloying of a ductile–ductile system [134].

Mechanical alloying (MA) is known as a solid state method to process the materials in the powder form at ambient temperature and produce MMNCs with an appropriate distribution of reinforcement particles and a fine microstructure [133]. In addition, MA is usually utilized to produce nanocomposites and alloys, which are not easy to fabricate through conventional techniques, such as casting. In MA technique, the powders are loaded into a high energy ball mill with balls. The deformed particles are subjected to a repeating fracturing, deformation, and welding processes. This milling process results in intimate mixing of the starting ingredient particles in the atomic scale, producing different supersaturated solid solutions (SSSs), amorphous metal alloys and metastable crystallites, and reduces the grain size to the nanoscale. In the case of ductile metal powder, the initial collision of ball–powder–ball leads to flattening and work hardening when they undergo cold welding and heavily mechanically deforming—the layered structure of nanocomposite particles is formed when they are brought into close contact. As can be schematically seen in Figure 3, this layered structure consists of different combinations of starting powders. The total energy of milling can be adjusted by changing the fraction of the weight of the balls/the powder, the design of media, the atmosphere of milling, speed, time and temperature of milling. During milling, in special cases, stearic acid or acrylic acid is introduced to the mixing media as an organic process control agent (PCA). In fact, the addition of PCA to the powder mixtures prevent the agglomeration due to the adsorption of PCA on the surface of particles, minimizing the cold welding between deformed particles. 

In order to disperse the bundled CNTs, the organic surfactant can be utilized alternatively, whereas in the case of graphene, no surfactant is necessary [135,136].

After the mixing, consolidation is the key step in the fabrication of MMNCs. One of the advanced powder-based manufacturing methods of MMNCs is the LPBF process, which is one of the most important AM processes [137]. In fact, in this technology, the MMNCs will be produced via either a powder bed system or powder feed system. Surprisingly, it is found that through the LPBF process, which is a powder bed system, it would be possible to produce complexly shaped MMNCs in a single step [122]. For instance, Zhang et al., compared the effect of CNTs’ addition with the effect of graphite addition on the mechanical properties of laser aided AM Inconel 625 nanocomposites [138]. In another work, Lin et al., produced a MMNCs reinforced by graphene via a hybrid manufacturing process, including layer-wise deposition of metal/graphene, followed by laser shock peening [31]. In general, the AM processes are recognized as better alternatives for the conventional production processes of MMNCs and in the future, will be one of the hot topics in this filed, and consequently, the number of publications on this topic will significantly increase [137,138,139,140].

**Figure 4:**
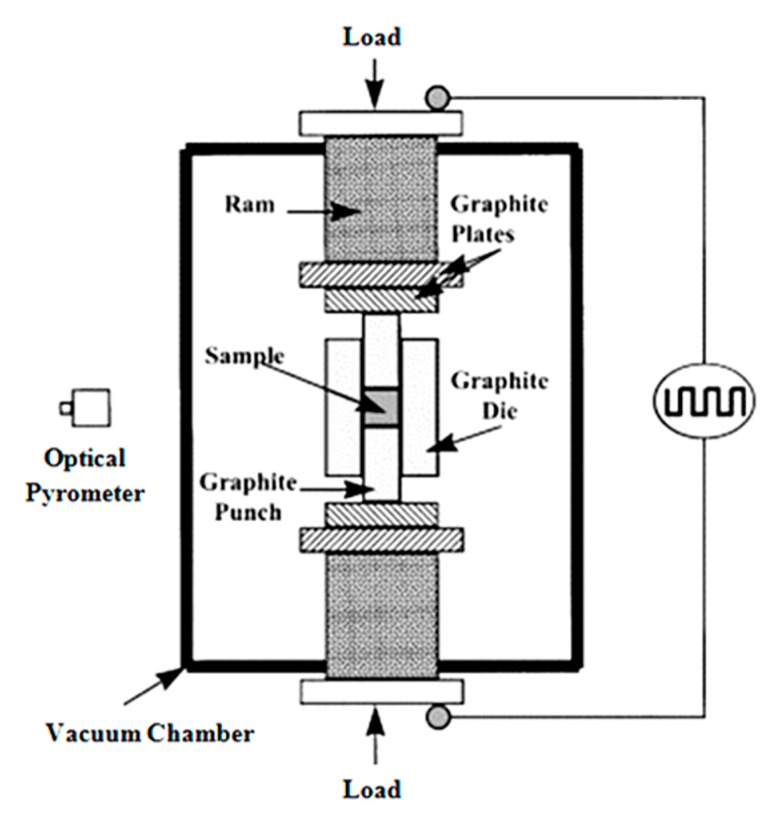
Schematic of spark plasma sintering [146] (Copyright Elsevier, 2000, Journal of the European Ceramic Society).

On the other hand, to obtain denser products through the conventional sintering, a long time of processing and a high temperature are required, whereas low-temperature processing and a short sintering time are offered by using SPS for consolidating nanocomposite powders [141,142]. As can be observed in Figure 4, in this process, powders are consolidated in a graphite die through the application of DC pulses and uniaxial pressing. Indeed, a local heat which is generated between particles as a consequence of a spark discharge leads to rapid heating, and then an increase of the sintering rate. In the case of nano-powders, which suffer from extensive grain growth during conventional sintering, SPS is particularly suitable for the consolidating of the powders. In the literature, several works have been devoted to fabricating graphene-reinforced nanocomposites by the SPS process [143,144,145].

**Figure 5:**
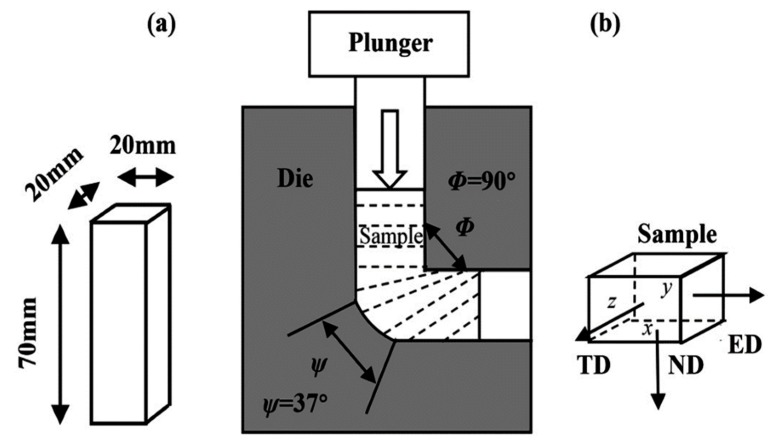
(**a**) The rectangular samples which are prepared for equal-channel angular pressing (ECAP); (**b**) ECAP process with reference axes, ED (extrusion direction), TD (transverse direction) and ND (normal direction) with respect to x-plane [150].

Moreover, it is possible to achieve ultrafine or submicron-grains in the MMNCs nanocomposites reinforced by graphene by using severe plastic deformation (SPD) processes, such as equal-channel angular pressing (ECAP) [147,148]. ECAP has some merits, such as the least load requirement, a constant cross-section and a large strain. In this technique, the press of MMCs is carried out by a die where two channels with the same cross-section, intersect at an angle Φ, as demonstrated in Figure 5. The sample is pressed by applying a plunge through the die in such a way that shear deformation occurs at the intersection of two lateral channels. Since the deformed cross-sections are similar to the starting ones, it is possible to implement a severe plastic deformation on the sample. During ECAP, a high density of dislocations is generated, that arrange accordingly into metastable sub-grains of high-angle grain boundaries [149].

## 6. Strengthening Mechanisms

In general, it is proven that in MMNCs, the strenghening effect can be obtained via different strengthening mechanisms, such as load transfer, Orowan looping, thermal expansion coefficient mismatching and Hall–Petch strengthening (grain refinement) [23,151,152,153].

The load transfer strengthenig effect is the first possible mechanism for strengthening in MMNCs. In this mechanism, load transfer is from the soft matrix to a high strength reinforcement, and this mechanism depends on the interface between the matrix and reinforcing particles. In principle, stronger interfacial bonding could result in high load transfer, and accordingly increase the final strength of the nanocomposite. However, it was reported that in the MMNs consisting of low reinforcement content, the load transfer effect does not contribute significantly in the strengthening of MMNCs [152].

Orowan looping is another important strengthening mechanism which is based on the restriction of dislocation movement by nano-scale reinforcement [154]. In fact, whenever a particle interacts with a dislocation, it undergoes a stress, and if it can withstand the force, the dislocation starts to bow, and finally an Orowan loop forms around the particle. In this mechanism, uniform dispersion of reinforcement plays an important role in order to achieve a full strengthening effect. Here, it can be noticed that by decreasing the space between the graphene nanoplatelets within the matrix (through a uniform dispersion) the strengthening effect through this mechanism would be significant [23].

In addition, the significant difference between the thermal expansion coefficient of metal matrices and reinforcing particles, in particular graphene, can result in the prismatic punching of dislocations at the interface, consequently leading to the strengthening of the nanocomposite [155].

Grain refinement is one of the significant effects of the reinforcement addition and it relies on the particle size and volume fraction on nanoparticles, so that the grain size decreases either by increasing the volume fraction of reinforcement or decreasing the size of reinforcement [156].

## 7. Metal Matrix Nanocomposites Reinforced by Graphene

### 7.1. Aluminum Matrix Nanocomposites Reinforced by Graphene

Aluminum matrix nanocomposites (AMNCs) are known as one of the main metal matrix nanocomposites, which commonly utilized in different applications, such as aerospace and transportation, while they seldom used for electronic packaging applications [157]. The superior properties of Al-matrix nanocomposites, like appropriate strength, electrical conductivity, low density, high thermal conductivity and low cost resulted in their broad applications. In this kind of MMNCs, carbon allotropes or some ceramic particles are employed as reinforcement materials, such as Al_2_O_3_, SiC, CNTs, diamond, graphene and graphite. In order to produce aluminum matrix nanocomposites, two common methods have been employed which are reinforced by particles on a large scale: One is powder metallurgy process (solid state), and another one is casting (liquid state). In the literature, several efforts have been carried out to produce AMNCs through powder metallurgy for investigating their mechanical characteristics [44,158,159,160,161]. In addition, during the last decade, there is a growing body of literature devoted to producing these MMCs that are reinforced by different nano and micron ceramic reinforcing materials, by means of various production techniques. For example, Al–Al_2_O_3_ and Al–SiC via powder metallurgy method and casting [162]; Al–Al_2_O_3_ by SPS [163]; Al nanocomposite-graphene by sintering and hot extrusion [39]; Al–B_4_C by SPS and reactive infiltration [164,165]; Al–MgO by stir casting and powder metallurgy [166]; Al–MWNT through ball milling, hot isostatic pressing and extrusion [38]; and Al–SiC through pressure-less infiltration, casting, powder metallurgy and extrusion process [167,168,169]. Nevertheless, a considerable amount of publications have been focused on describing the role of various allotropes of carbon on mechanical features of the aluminum matrix nanocomposites.

Since the strengthening effect of various reinforcements on AMNCs is a key factor in their structural applications, extensive research has focused on this subject (as can be seen in Table 1). In Al and Cu alloy based nanocomposites, the CNTs and graphene increases the strengths and tribological features of the nanocomposites. It has benn found that CNTs and graphene refine the grains in aluminum alloys in such a way that leads to additionally higher strength. Furthermore, the effectiveness of pure aluminum increases in the presence of carbon nanotubes and graphene [170]. Graphene presents excellent tensile strength and toughness, 130 GPa and 0.5–1 TPa, respectively, so when it is introduced, it is an efficient reinforcement to strengthen and stiffen the metal. The concentration of graphene and the sintering environment has a remarkable effect on mechanical features. By embedding of GNPs to the metal matrix nanocomposite, due to the density differences, the density of matrix nanocomposite decreases. In AMNCs, the measured experimental density of both pure aluminum and nanocomposites are higher than the theoretical ones. It can be attributed to the oxidation of Al in such a way that causes the formation of high-density aluminum oxide during the sintering process [171]. There is a noticeable mismatch between the coefficient of thermal expansion of the matrix nanocomposite and graphene that resulted in prismatic hitting of dislocations at the boundaries and the outstanding strengthening of the nanocomposite matrix. The density of dislocations depends on the range of reinforcement particles. The high density of dislocation resulted in increasing of the nanocomposite strength [58]. However, so far, enough attention has not been paid to the effect of graphene as a superconductive reinforcement on the thermal behaviour of AMCs. 

**Figure 6:**
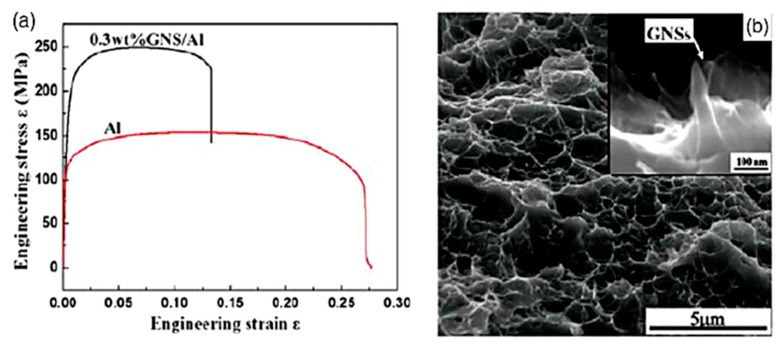
(**a**) Tensile characteristics of Al–0.3 wt.% graphene nanosheet (GNS) nanocomposite and the Al sample; (**b**) fracture surface of Al–0.3 wt.% GNS nanocomposite; the inset shows the graphene nanoplatelets (GNSs) pulled out [39] (Copyright Elsevier, 2012, Scripta Materialia).

On the other hand, according to the authors’ knowledge, so far the powder metallurgy method is known as the only technique to successfully fabricate the graphene reinforced aluminum matrix nanocomposite [38,39]. For this purpose, the compressibility and sinterability of Al nanocomposites reinforced by graphene nanoplatelets (GNPs) have been studied by Saboori et al. [172,173]. According to their results, at lower compaction pressures, less than 500 MPa, the mechanism of consolidation for Al–GNP nanocomposites is particle arrangement, whereas, at higher compaction pressures, more than 500 MPa, it is plastic deformation of particles. Furthermore, they found that both the compressibility and sinterability of nanocomposites remarkably decrease with increasing the GNP content. Pérez-Bustamante et al. [46] have synthesized Al–GNP nanocomposites through the mechanical alloying technique followed by a conventional press-sintering process. Their results revealed that by increasing the graphene content, the hardness of nanocomposite increases significantly. Wang et al. [39] reported that the tensile strength of AMC was enhanced about 62% by incorporating 0.3 wt.% graphene nanosheets (GNSs) to the nanocomposite as a reinforcement (Figure 6a). In addition, the fracture surface of the aluminum nanocomposite is shown in Figure 6b. As can be seen in the fracture surface of Al–GNS nanocomposites, there is a crack initiation point in at the interface of matrix/reinforcement and consequently, this weakness leads to a lower ductility. In another work, Bartolucci et al. [38] found that by the addition of 0.1 wt.% GNPs to aluminum matrix nanocomposites, the tensile strength of nanocomposite increased, while sacrificing the strain at failure of the nanocomposite in comparison with the pure aluminum matrix.

**Figure 7:**
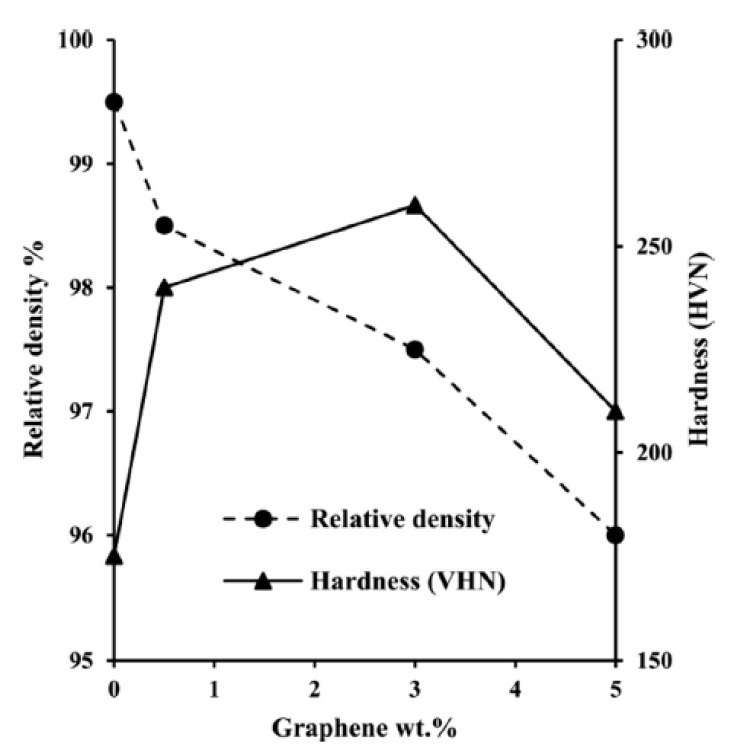
Variation of relative density and hardness in AA2124 nanocomposite as a function of the addition of graphene as a reinforcement [170] (Copyright Elsevier, 2015, Composites Part B: Engineering).

Recently, Shao et al. [174] have used graphene oxide (GO) and graphene nanoplates as reinforcement in 5083Al-matrix nanocomposites produced by pressure infiltration method. Based on the XRD analysis, there was no peak related to Al_4_C_3_ in the patterns of different graphene. Although in both of the nanocomposites needle-like Al_4_C_3_ was observed, but the content of Al_4_C_3_ phase in the GNPs-5083Al nanocomposite was much lower than GO-5083Al. Moreover, in the GNPs-5083Al nanocomposite, the segregation of magnesium element observed at the surface of the graphene nanoplates which mentioned to the prevention effect of magnesium element on the formation of Al_4_C_3_ phase. Their experimental results showed that through the addition of GO and GNPs, the yield strength of both nanocomposites has been slightly improved. Furthermore, the tensile strength of the GNPs-5083Al nanocomposite increased by 14%. In other research, Ghazaly et al. [175] have produced graphene-Al by using various weight percentages of graphene (0.5, 3 and 5 wt.%) using PM process followed by uniaxial compaction at room temperature and hot extrusion. They revealed that by increasing the amount of graphene, there was a decrease in the density of nanocomposites (Figure 7). Moreover, their results showed that (Figure 7), the introduction of graphene up to 3 wt.% causes to the enhancement of nanocomposite hardness (about 47.5%) in comparison with unreinforced AA2124 alloy.

**Figure 8:**
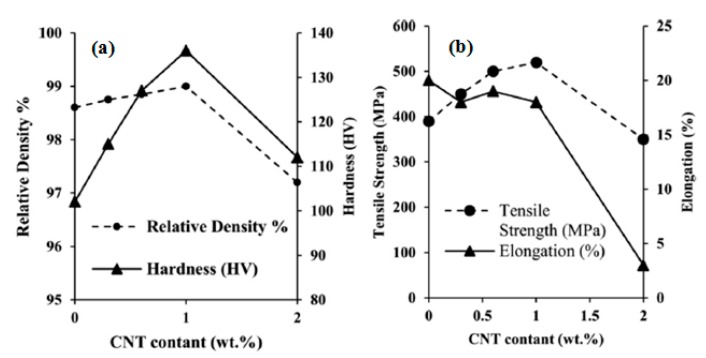
(**a**) Relative density and hardness. (**b**) Tensile strength and elongation as a function of the carbon nanotube’s (CNT’s) content for AA2024-MWNT nanocomposites [170] (Copyright Elsevier, 2015, Composites Part B: Engineering).

While, the hardness reduced by further increasing the graphene content (up to 3 wt.%) although, its values were more than the monolithic alloy and less than the reinforced nanocomposite with 0.5–3 wt.% graphene. By comparison between Figure 7 and Figure 8, it is clear that graphene-Al presents superior mechanical characteristics compared to CNT-Al.

**Figure 9:**
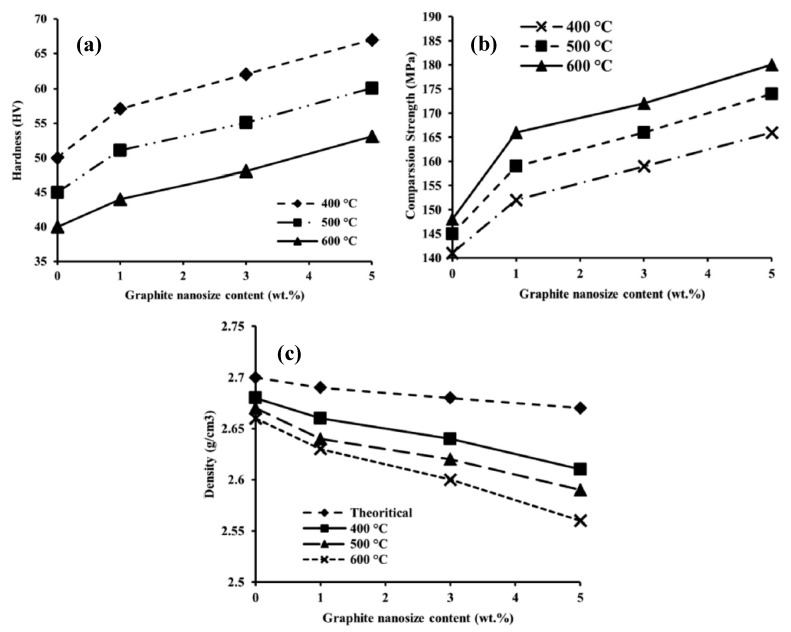
Variation of (**a**) Vickers hardness, (**b**) compressive strength and the (**c**) density of graphene-Al nanocomposites with various percentages of exfoliated graphite nanoplates at different sintering temperatures [176] (Copyright Elsevier, 2012, Journal of Industrial and Engineering Chemistry).

The variation of thermal conductivity and the Vickers hardness of Al-GNPs nanocomposites in the presence of graphene have been studied by Saboori et al. [172]. They reported that at low graphene content, the thermal conductivity is higher than the nanocomposites with a higher graphene content. These results can be explained by the formation of GNPs agglomeration at higher graphene content. In addition, by increasing the graphene content, there is a noticeable trend in the increment of the Vickers hardness. However, it should be noted that the deterioration of the Vickers hardness happens at a higher GNPs content owing to the agglomeration of GNPs. Latief et al. [176] have fabricated graphene-Al nanocomposites utilizing various percentages of exfoliated graphite nanoplates through powder metallurgy method to investigate the physical and mechanical characteristics of nanocomposites. They found that by increasing the amount of graphene up to 5 wt.%, the Vickers hardness (Figure 9a) and compression strength (Figure 9b) increases whereas, the density decreases (Figure 9c).

Recently, Saboori et al. [177] have fabricated GNPs–Al uminum by conventional powder metallurgy and hot rolling to compare the microstructure and thermal conductivity of the nanocomposites produced through both methods. The number of GNPs agglomerates significantly increased by increasing the content of graphene. By introducing 1 wt.% GNPs to aluminum nanocomposites, all the GNPs were located at the grain boundary so that the grain size reduced to 6 μm. Moreover, due to the presence of pores at the grain boundaries, the interface between the matrix and reinforcement was not strong enough to enhance the final properties. It should be underlined that in the case of GNP–Al nanocomposites synthesized by hot rolling, despite the presence of external forces during the shaping and consolidation, there was still some porosity at the interface of aluminum and the GNPs. Regarding the thermal conductivity measurements, due to the graphene clustering and weak interfacial bonding, the thermal conductivity of GNPs–Al nanocomposites increased slightly, followed by a dramatic decrease. To compare the models and practical outcomes, the experimental results were found, interestingly, in the range of the parallel model. This matter proves the occurrence of a preferred orientation of GNPs within the matrix during the consolidation. The high thermal conductivity of samples fabricated through hot rolling can be related to their higher density and lower porosity content, despite the preferred orientation of GNPs and GNPs’ agglomerate formation. In other work, the effect of GNPs on the microstructure and mechanical performance of AlSi10Mg/GNPs nanocomposites were investigated (by Saboori et al. [178]). In that study, 0.5 and 1 wt.% GNPs–Al Si10Mg composites were fabricated by a wet mixing technique, followed by two-step consolidation, including hot compaction and then hot extrusion at 400 °C. Their results showed that the addition of GNPs (more than 0.5 wt.%) to the AlSi10Mg nanocomposite caused the agglomeration of GNPs, so introduce the internal porosity in the nanocomposite in such a way that lead to the deterioration of the mechanical properties of the nanocomposite, while the hardness and tensile strength of Al alloy matrix improved slightly by the addition of 0.5 wt.% of GNPs. Guan et al. [179] produced graphene-aluminum nanocomposite by the incorporation of graphene-cupper powder into melted aluminum, followed by stirring and cooling it to ambient temperature. Based on their results, the graphene clustered intensely, which decreases the strengthening effect of GNPs. On the other hand, the hardness of GNPs-reinforced Al nanocomposite increased by about 40% in comparison with the pure Al. Despite the first investigations on graphene-Al matrix nanocomposites, which reported decreases of strength and hardness of nanocomposite, the rest of the researchers had positive results in this field. Due to the formation of Al_4_C_3_, as a consequence of poor controlling of the early synthesizing conditions, intensive deterioration of the mechanical characteristics of the nanocomposites occurred [32].

Lately, 1 wt.% GNPs reinforced 2009Al nanocomposites were synthesized via a combination of PM and multi-pass friction stir processing (FSP) by Zhang et al. [49]. Their microstructural evaluations showed that, as the number of FSP passes increased, the distribution of GNPs remarkably improved, in such a way that after two passes a uniform dispersion of GNPs was obtained. Compared with the 2009 Al alloy, a better elongation, a maximum ultimate tensile strength and yield strength was achieved: Up to 10%, 23.3% and 30.5%, respectively, after the two-pass FSP. In FSP nanocomposites, GNPs with layered structures were well retained, and most of the interfaces of GNP–Al were clean and well bonded. By increasing the number of FSP process, from one to four passes, initially, the strengths and the elongation of the nanocomposites enhanced, and thereafter declined. The calculation of strength based on the load transfer effect showed that the high efficiency of strengthening was related to the large specific surface area of homogeneously dispersed graphene. Moreover, Wejrzanowsky et al. numerically and experimentally studied the potential and limitations of single-layer graphene and multi-layer graphene to be used to improve the thermal conductivity of copper [180]. In line with the other findings in the literature, they also found that the quantity, size, orientation and distribution of graphene markedly affect the thermal conductivity of MMNCs. 

### 7.2. Magnesium Matrix Nanocomposites Reinforced by Graphene

**Figure 10:**
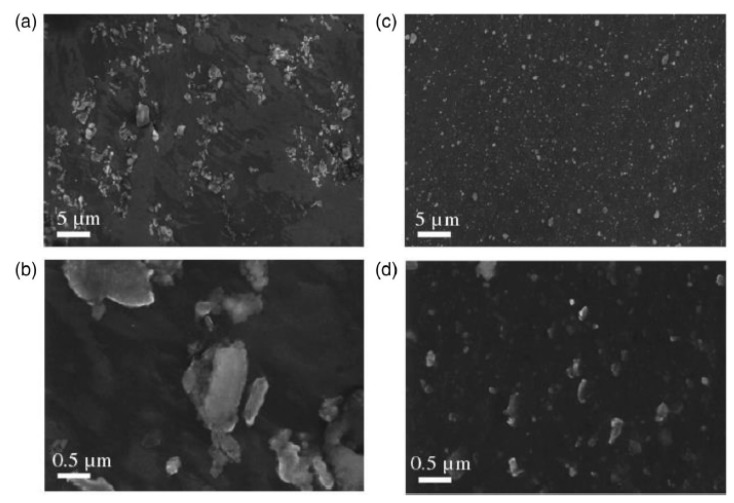
(**a**,**b**) SEM micrograph of the as-cast ultrasonic processed plate of the GNPs incorporated in the Mg matrix and (**c**,**d**) the ultrasonic processed and solid state stirred sample, at low and high magnifications [61] (Copyright Elsevier, 2012, Scripta Materialia).

**Figure 11:**
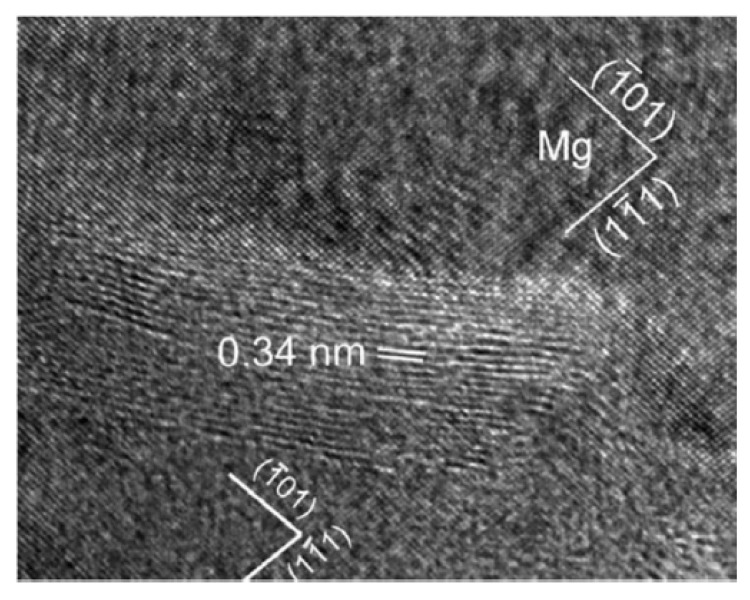
High-resolution transmission electron microscopy (HRTEM) micrograph of the GNPs incorporated in the Mg matrix [61] (Copyright Elsevier, 2012, Scripta Materialia).

Chen et al. [61] reported the first outcomes on GNPs-reinforced Mg matrix nanocomposites. They employed liquid state ultrasonic processing and solid-state friction stirring to synthesize the nanocomposite. SEM and TEM images of this nanocomposite are shown in Figure 10 and Figure 11, respectively. As is shown in Figure 10a, the GNPs are well embedded in the magnesium matrix. Nevertheless, due to the existence of micrometre-sized agglomerates in the Mg matrix (Figure 10a,b), the dispersion of the GNPs needs to be improved. Uniformly distribution (Figure 10c,d) of GNPs in the Mg-matrix confirmed that a combination of those two techniques could successfully insert and disperse the GNPs into the Mg-matrix to produce bulk MMNCs. 

In addition, as can be observed in the TEM image (Figure 11), for GNPs with an interplanar distance of 0.34 nm incorporated in the magnesium matrix, porosities and reaction products were not detected. Consequently, very good bonding should exist between the GNPs and the Mg matrix. Furthermore, a TEM image shows the same orientation of the Mg-matrix around the GNPs, which demonstrated the GNPs were embedded inside a magnesium grain. Under the same conditions, the microhardness of the (1.2 vol.%) GNPs-Mg nanocomposite improved by about 78% compared that to the pure Mg which had values of 66 and 37 kg/mm^2^. They have also reported that graphene nanoplatelets have a significant strengthening effect on the Mg-matrix nanocomposite [61].

**Figure 12:**
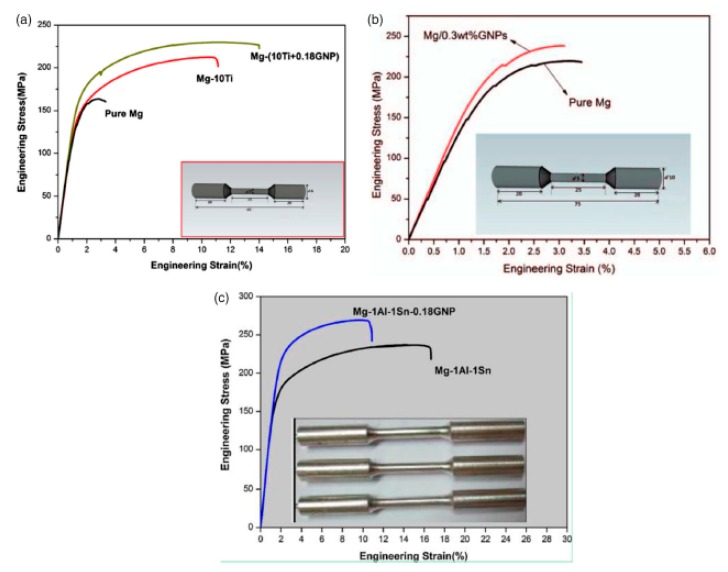
(**a**) Tensile test of pure Mg, Mg–10Ti alloy and Mg–(10Ti + 0.18 wt.% GNPs) nanocomposites at room temperature, (**b**) tensile test of pure Mg and 0.3 wt.% GNPs-Mg nanocomposite, and (**c**) tensile test of 0.18 wt.% GNPs-Mg–1%Al–1%Sn nanocomposite and Mg–1%Al–1%Sn alloy [32] (Copyright, 2016, Materials Science and Technology).

Rashad et al. [58,59,63,181,182] have fabricated an Mg-(10Ti + 0.18 wt.% GNPs) nanocomposite, a (0.3 wt.%) GNPs-Mg nanocomposite and a 0.18 wt.% GNPs-Mg–1%Al–1%Sn nanocomposite through semi-powder metallurgy followed by hot extrusion. In the case of Mg-(10Ti + 0.18 wt.% GNPs) nanocomposite, the incorporation of graphene nanoplates causes the enhancement of ultimate tensile strength, ductility and yield strength (Figure 12).

This improvement of ductility in that work was explained by the level of porosity in the fabricated pure Mg and nanocomposites [181]. It is also found that the brittle characteristic of pure Mg is because of its limited number of slip systems in Mg crystals [59]. The hardness, yield strength and ultimate tensile strength of 0.3 wt.% GNPs-Mg nanocomposite were 68.5 HV, 197 and 238 MPa, respectively, which presented a slight increase in the mechanical characteristics in comparison with the pure magnesium. In addition, yield strength and ultimate tensile strength of 0.18 wt.% GNPs-Mg–1%Al–1%Sn nanocomposite increased by 29.2% and 14% with respect to the pure Mg. Based on their report, graphene acts as a reinforcement for Mg matrix nanocomposites and this claim was confirmed by the improvements in their mechanical properties.

**Figure 13:**
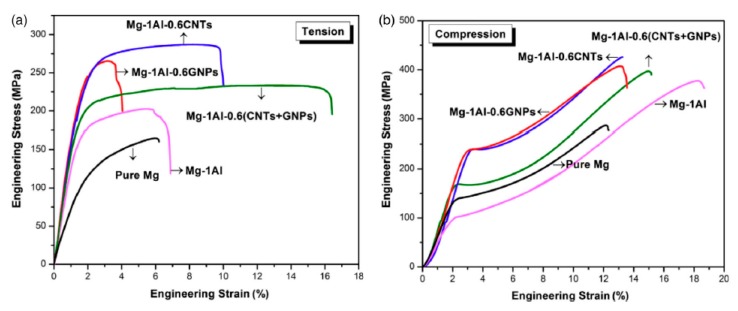
(**a**) Tensile and (**b**) compression stress–strain curves for pure magnesium and its nanocomposites at room temperature [63] (Copyright Elsevier, 2014, Journal of Alloys and Compounds).

**Figure 14:**
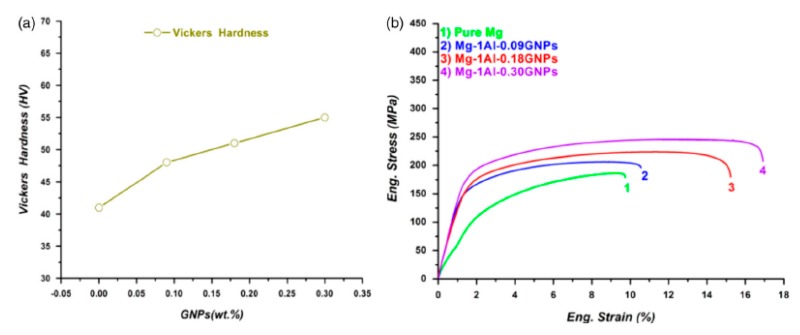
Mechanical properties of pure Mg and its nanocomposites at room temperature: (**a**) Vickers hardness, and (**b**) tensile stress–strain curve [59] (Copyright Elsevier, 2015, Materials Science and Engineering: A).

The effect of carbon nanotubes and graphene on the mechanical characteristic of pure Mg have been studied by Rashad et al. [63]. They revealed that, in the presence of CNTs or graphene or (CNTs + graphene) with the same ratio, the best strengthening effect was presented by CNTs. However, CNTs + graphene nanocomposites showed the best ductility and a good strengthening impact, as can be observed in Figure 13. Moreover, in other research [59], they have synthesized GNPs-reinforced Mg matrix nanocomposites through powder metallurgy processing. They found that, with the increasing of the content of graphene, the hardness, yield strength and fracture strain of the nanocomposite all improved (Figure 14). 

**Figure 15:**
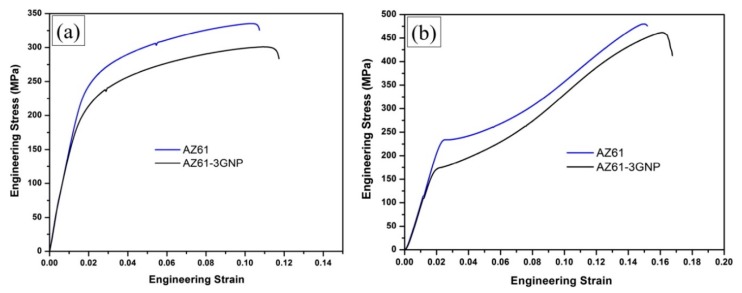
(**a**) Tensile, (**b**) compression stress–strain curves of AZ61 alloy and its nanocomposite at room temperature [183] (Copyright Elsevier, 2016, Materials & Design).

In another work [183], AZ61 magnesium alloy reinforced by GNPs has fabricated by an integrated melt deposition method. In this investigation, because of the uniform distribution of GNPs within the matrix, they exhibit a significant influence on the grain refinement and changing in basal textures, which resulted in a marked improvement in the mechanical performance of nanocomposites at room temperature (Figure 15). As can be observed in Figure 15a, yield strength and ultimate tensile strength of nanocomposite exhibited 26% and 11.7% increases in comparison with monolithic alloy. Based on Figure 15b, the ultimate compressive strength and compressive yield strength of nanocomposite were enhanced up to 4.12% and 32.9%, respectively, with inconsiderable decreases in compressive fracture strain values. Furthermore, nanocomposite with 3 wt.% GNPs showed a 15.5% increase in Vickers hardness, compared to the pure AZ61 alloy. During those experiments, the tensile strength of as-extruded GNPs-AZ61 nanocomposite was demonstrated, in the range of 75 °C to 225 °C. Experimental results revealed that, by increasing the testing temperature, total fracture strain increased, and tensile yield strength decreased. The increase of fracture strain (at high temperature) can be explained with the remarkable grain refinement and uniform distribution of particles.

**Figure 16:**
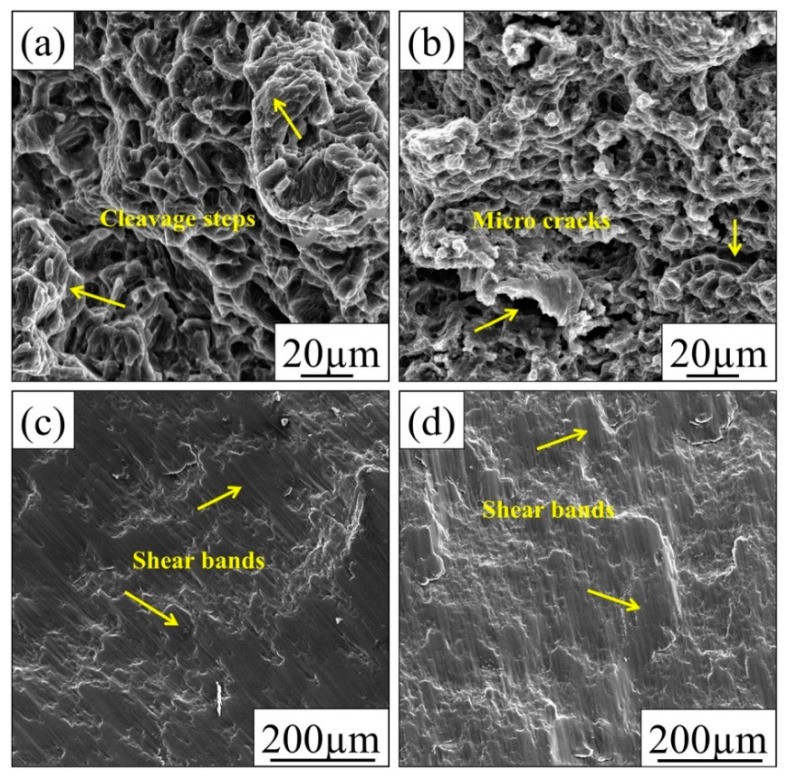
Tensile fracture surface of extruded (**a**) AZ61 alloy, (**b**) AZ61–3GNP nanocomposite; and compressive fracture surface of extruded (**c**) AZ61 alloy and (**d**) AZ61–3GNP nanocomposite [183] (Copyright Elsevier, 2016, Materials & Design).

Figure 16 demonstrates the fracture surface of fabricated AZ61 alloy and its nanocomposite after tensile and compressive tests. The tensile fracture surface image of AZ61 alloy (Figure 16a) shows a mixed mode fracture with cleavage steps, which is evidence of ductile material. Likewise, the tensile fracture surface of nanocomposite (Figure 16b) demonstrates a significant deformation, along with several microcracks and cavities that could be responsible for the reduction of fracture strain values in comparison with AZ61alloy. Moreover, the existence of microcracks in the fracture surface of the nanocomposite can be explained by high dislocation densities at the interface of GNP/matrix. According to compressive fracture analysis, there were shear bands in pure AZ61 alloy and its nanocomposites, as illustrated in Figure 16c,d. The presence of these shear bands can be related to work hardening behaviour and heterogeneous deformation, because the rate of work hardening is higher for samples failed by shear bands. Regarding the compressive loading, all fractures occurred at a 45° angle.

Recently, Meng et al. [62] produced GNPs–Mg laminated nanocomposites. They showed that by increasing GNPs from 0.25 vol.% to 0.75 vol.%, the tensile strength of the nanocomposite increases to 160 and 179, respectively, compared to pure Mg (136 MPa). The load transfer capacity improved due to the uniform distribution of graphene and constrained the transformation of Mg foils to monolithic materials. The motion of dislocation was prevented, as a consequence of the induced laminated structure, and so strengthened the nanocomposites. In other work, Du et al. [65] fabricated GNPs-magnesium alloy (ZK60) nanocomposites using the melt stirring and hot extrusion processes. For this purpose, graphene was mixed with magnesium powder and extruded into the rods. Thereafter, the extruded rods were used as a precursor for melting, and that combination of processes effectively guaranteed the dispersion of GNPs. In comparison with ZK60 alloy, in the presence of only 0.05 wt.% GNPs, the yield strength of the nanocomposite increased by 62%.

As reported in the literature, Mg-Al-GNPs nanocomposites showed better mechanical characteristics in comparison with the Mg-Al-CNT nanocomposites. These improved mechanical characteristics were explained by the high specific surface area, better nanofiller matrix adhesion ascending from the crumpled surface and the 2D structure of graphene nanoplatelets [184]. Moreover, the 1D structure of CNTs results in the poor dispersion of CNTs in the metallic matrix and agglomeration that results in the lower mechanical performances [185].

**Figure 17:**
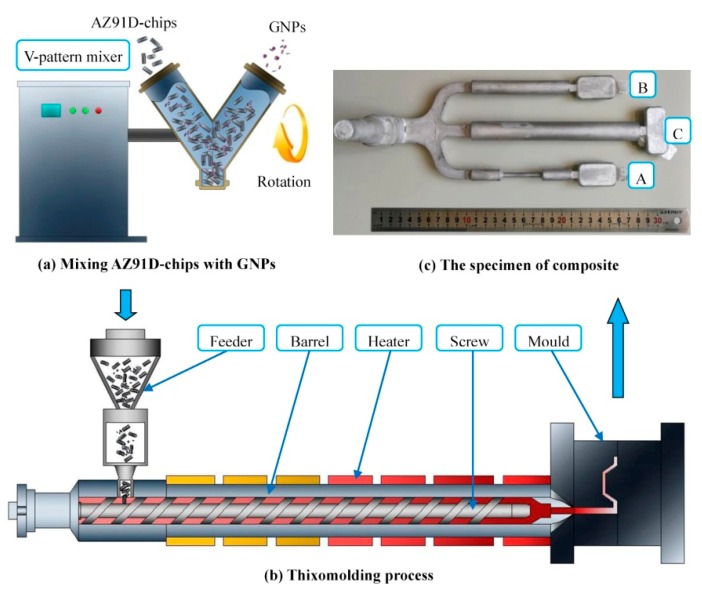
The schematic diagram for the preparation of GNPs–AZ91D magnesium alloy [186] (Copyright Elsevier, 2019, Journal of Alloys and Compounds).

For the first time, in 2019, AZ91D Mg alloy-GNPs nanocomposites were produced via a thixomolding process (Figure 17) by Chen et al. [186]. They revealed that there was a strong interface between AZ91 and GNPs, and that GNPs were well-dispersed in the Mg matrix. The obtained results indicated that the optimal Ultimate Tensile Strength (UTS), elongation, Vickers hardness and porosity content of nanocomposites were 38.4%, 85.7%, 29.9% and 33.3% higher than those achieved for AZ91D alloy, respectively. In addition, they reported that the thixomolding process is a very promising technique for mass-scale production.

### 7.3. Copper Matrix Nanocomposites Reinforced by Graphene

**Figure 18:**
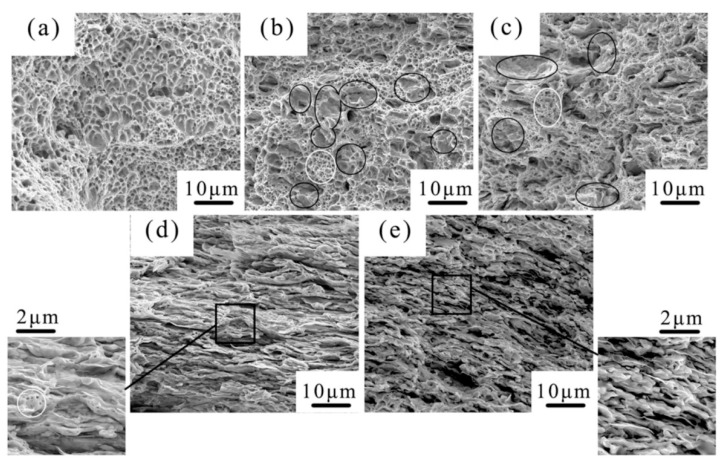
Surface images (SEM) of pure Cu and GNPs–Cu nanocomposites: (**a**) Pure Cu, (**b**) 0.2 vol.% GNPs–Cu, (**c**) 0.8 vol.% GNPs–Cu, (**d**) 2.0 vol.% GNPs–Cu and (**e**) 4.0 vol.% GNPs–Cu [53] (Copyright Elsevier, 2016, Carbon).

Graphene-reinforced copper matrix nanocomposites were produced approximately at the same time as graphene-reinforced aluminum matrix nanocomposites. Chen et al. [53] fabricated GNPs–Cu by molecular-level mixing method, followed by the SPS process to evaluate the microstructure, mechanical properties, thermal and electrical conductivity and wear properties of the nanocomposites. They revealed that the dispersion of GNPs in the nanocomposite is influenced by its content. In fact, in the presence of low GNPs concentration, the GNPs dispersed randomly. Moreover, it was found that when GNPs’ concentration was above 2.0 vol.%, they were oriented perpendicularly to the consolidation force during the SPS process. The fracture surfaces of pure Cu and GNPs–Cu nanocomposites are shown in Figure 18, which confirms the homogeneous dispersion of GNPs in the Cu-matrix, even in the presence of 4.0 vol.% of GNPs. This homogeneous dispersion of GNPs significantly prohibited the negative influence of GNPs agglomerations on the GNPs–Cu nanocomposites’ characteristics. With increasing the content of graphene, both size and the depth of the dimple kept decreasing; that is a sign of brittleness of the nanocomposites. Some graphene was present completely on the fracture surface (black circled areas in Figure 18b,c) which was related to the propagation of cracks along the interface of phases after interface debonding. Moreover, as the graphene content increased, the mechanical characteristics of copper strengthened, though strengthening effects increased firstly and then decreased as a consequence of graphene enhancement. Their results also showed that, by increasing the amount of graphene, thermal diffusivity has a constant decline. In addition, the negative effect on electrical conductivity as a result of graphene incorporation was minor, whereas the tribological performance improved significantly.

**Figure 19:**
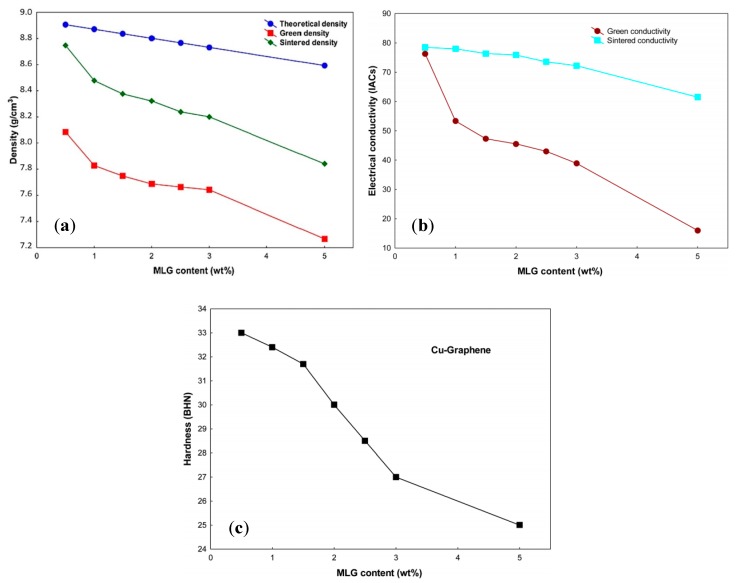
The effect of multilayer graphene (MLG) content on the (**a**) density, (**b**) electrical conductivity and (**c**) hardness of MLG–Cu nanocomposites [188] (Copyright Springer Nature, 2015, Metals and Materials International).

Li et al. [187] studied the mechanical features of Ni-nanoparticle-decorated graphene nanoplatelets (Ni–GNPs) as a reinforcement in the copper matrix (Ni–GNPs–Cu). Ni nanoparticles well distributed and firmly adhered on the surface of GNPs. Regarding the monolithic Cu, a considerable improvement in UTS (about 42%) was observed in the presence of only 0.8 vol.% Ni–GNPs. This increase can be related to the unique structure of Ni–GNPs that can cause good distribution and strong interfacial bonding of GNPs–Cu. Nevertheless, due to the clustering of GNPs, the UTS of 0.8 vol.% GNPs–Cu nanocomposites was lower than that of the pure Cu. Their results showed that Ni–GNPs are new and useful reinforcements to improve the mechanical characteristics of the graphene-metal nanocomposites. In another study, Varol et al. [188] investigated the effect of multilayer graphene (MLG) addition on the green and sintered characteristics of copper nanocomposites using flake powder metallurgy, as a new production method. As can be observed in Figure 19a, with increasing the MLG content, sintered density decreased due to the increase in the content of agglomeration which resulted in the prevention of particle–particle contact during the sintering process. For the monolithic Cu specimen, the green density was 16.4% higher than that of 5 wt.% MLG–Cu nanocomposites. By incorporating 0.5 wt.% MLG a high conductivity value achieved, of about 78.5 IACs. Whereas, after sintering the electrical conductivity of 5 wt.% MLG–Cu nanocomposites was 61.48 IACs (Figure 19b). By embedding of MLG (>3 wt.%), the decreasing rate of hardness considerably increased (Figure 19c), which can be explained by the reducing density and nonhomogeneous distribution of MLG particulates in the copper matrix.

The mechanical and wear characteristics of copper-based nanocomposites containing varying content of graphene nanosheets (GNSs) and graphite (Gr) have been compared by Li et al. [189]. They found that (Table 6), the GNSs-Cu nanocomposites showed a higher relative density, bending strength and microhardness regarding the Gr–Cu nanocomposites in the presence of the same volume fraction of reinforcements. With Gr incorporation, limited effects were found for the reduction of friction and wear, whereas GNSs addition showed a significant decrease in the friction coefficients and wear rates. They believed that, higher mechanical and wear performances of GNSs–Cu nanocomposites could be related to the unique strengthening impact of GNSs when compared with those of Gr. Those results showed that GNSs acts as an impactful lubricant and also a desirable reinforcement, which makes them an ideal reinforcement for copper matrix nanocomposites. Zhang et al. [190] used two different kinds of graphene derivatives (GNPs and reduced graphene (RGO)) to produce Cu matrix nanocomposites via a modified molecular-level mixing technique. Based on the microstructure results, the GNPs presented themselves as flake-shaped, while the RGO presented irregular strip or sheet shape in the nanocomposite. After sintering, the two reinforcements well adhered with the Cu-matrix. Although 1 vol.% RGO still homogeneously distributed in the Cu-matrix, GNPs demonstrated an evident trend to aggregate when their content was above 0.5 vol.%. Moreover, they reported that GNPs and RGO exhibited various strengthening influences on Cu-matrix nanocomposites. At GNP contents below 0.5 vol.%, it exhibits excellent strengthening efficiency, whereas increasing the content of RGO from 0.5 to 1.0 vol.% it showed better strengthening efficiency.

Recently, other researchers [191] have fabricated MLG–Cu nanocomposites through molecular-level mixing combined with vacuum hot-pressing (VHP). MLG dispersed in the Cu-matrix with a preferred orientation, with the in-plane surface perpendicular to the hot-pressing direction. This preferred orientation resulted in considerable anisotropy in the thermal features of MLG–Cu nanocomposites. By raising the temperature, the length of the MLG bond gradually increased in the in-plane direction. In contrast, the MLG bond length in the out-plane directions, at first remarkably incremented, and then decreased at higher temperatures. The bond length variations for multilayer graphene are controlled by the CTE anisotropy of the MLG–copper nanocomposites in the same direction.

Since copper and graphene nanoplates have various densities, one of the issues to the homogeneous distribution of GNPs in the Cu-matrix is the production of GNPs–Cu nanocomposites. For this reason, in 2019, Shao et al. [144] fabricated GNPs–Cu nanocomposites via electrostatic self-assembly and spark plasma sintering. They reported that the load-transfer efficiency of GNPs in the nanocomposites improved with the two-dimensional structure flake copper powders. In addition, graphene nanoplates were uniformly adsorbed on the surface of copper powder by using the production techniques. The mechanical characteristics of the nanocomposites are shown in Table 7. As can be observed, compared to pure copper, by incorporating of 0.2 wt.% GNPs, the tensile strength and Vickers hardness of the nanocomposites were enhanced by 27% and 19%, respectively.

Some researchers compared the electrical conductivity of GNPs-copper nanocomposites after the hot isostatic press and as-sintered ones. They revealed that the electrical conductivity of nanocomposites considerably increases after HIP, which causes a decrease of the void content of GNPs–Cu nanocomposites. The electrical conductivity of GNPs–Cu nanocomposites produced by various methods is summarized in Table 8. It is clear that the process of fabrication, type quality and the weight percentage have a significant effect in the electrical conductivity of Cu nanocomposites.

### 7.4. Other Metal Matrix Nanocomposites Reinforced by Graphene

A noticeable amount of research has been aimed to investigate the effect of embedding graphene into the MMCs. The main applications of those nanocomposites, including their use as photo-catalysts, and in transformation materials, biosensors, energy storage, and so on. Those metal matrix nanocomposites consist of Ag-doped graphene-silver matrix nanocomposites [194], graphene (3D-GNs)–Ni nanocomposites [195], graphene (MLG)–Ti nanocomposites [196], graphene (GNPs)–Inconel 718 [197], graphene (MLG)–Ni_3_Al alloy [198], graphene–Fe [184], GNNs–Ag–Cu–Ti nanocomposites [199] and others. Some characteristics of those graphene-metal matrix nanocomposites are summarized in Table 9.

## 8. Potential Applications of Graphene-reinforced MMNCs

In recent years, MMNCs have been considered potential materials to be employed in different sophisticated industrial applications. Therefore, more research and learning has been carried out to explore the advances in their structures and possible applications. Indeed, over the last decade, a growing body of literature on MMNCs has shown that these materials, in particular reinforced by graphene, show great potential to be used in a range of engineering applications and lightweight alloys. Indeed, owing to their unique properties, such as high strength and toughness at high temperatures and low density, MMNCs are recognized as favourable materials to be employed in applications where conventional engineering materials, such as steel, are used. Although MMNCs often have low ductility and interior fracture toughness, they present remarkably higher stiffness and mechanical strength compared to matrix alloys. Therefore, selecting suitable reinforcement, characteristics and production techniques play vital roles in the resulting MMNCs [204].

As mentioned earlier, graphene-reinforced MMNCs have shown excellent features, such as high specific strength, good wear resistance, good thermal conductivity, excellent mechanical and optical properties, low thermal expansion coefficients and excellent corrosion resistance. Consequently, these nanocomposites are mainly utilized to make multifunctional products, and are used in some industries, including the automobile, sports, aerospace, chemical and electronics industries. For example, copper is the most suitable material to be employed in high-end applications like integrated circuits, electric switches and electronic packages, mainly due to its unique properties like high electrical and thermal conductivity, fatigue resistance, workability and corrosion resistance. Nonetheless, its poor mechanical properties, together with its high CTE limit its applications. Salvo et al. and Saboori et al. have shown that through the addition of graphene it would be possible to simultaneously enhance the mechanical, thermal and electrical conductivities of the Cu/GNPs nanocomposite, while reducing its CTE, making this nanocomposite the most suitable material to be used in the electric switches and electronic packages [3,192]. Dori Moghadam et al. have reported that due to the unique self-lubricating of MMNCs reinforced by graphene, they can be used in the fabrication of cylinder liners, pistons and gear surfaces [11]. Table 10 summarizes an overview of the potential applications of MMNCs reinforced by graphene.

Despite all the efforts that have been undertaken to develop new nanocomposite materials reinforced by graphene, still the production of these new materials in the industrial scale is faced by several challenges, like the uniform dispersion of graphene within the metallic matrix and interfacial bonding between graphene/metallic matrix.

## 9. Conclusions

In this review paper, we have thoroughly reviewed recent research into the development in the powder-based production, property characterization and application of magnesium, aluminum, copper, nickel, titanium and iron matrix nanocomposites reinforced with graphene. According to the review results, the conclusions can be drawn as follows:

- Owing to the unique characteristics, such as a high aspect ratio, an exceptional high elastic modulus and strength, and superior electrical and thermal conductivity, graphene could attract more attention to be utilized in the production of MMCs as a reinforcement to improve their mechanical, thermo-physical and wear properties.
- It is found that the production of these nanocomposites is faced with several challenges, such as the distribution of graphene, undesirable reaction, poor interfacial bonding and preferred orientation of graphene during the fabrication step.
- It is very interesting to point out that, according to the literature, some of the issues can be related to the nature of starting materials, whereas the rest can be attributed to the production process. Those related to the production process can often be addressed by altering the fabrication technique or by employing post-processing methods. More problematic, are the issues attributed to the matrix’s composition and reinforcement composition, its stability and the distribution of particulates, especially if they are in the nano-scale range.
- The majority of the literature has been concentrated on the production, and some studies have targeted the mechanical performance, thermophysical and self-lubricating characteristics.
- AM techniques, that are layer-wise manufacturing processes, are rapidly growing in the fabrication of MMNCs.
- In general, it is revealed that the mechanical performance of MMCs was significantly improved through the addition of graphene.
- The in-depth microstructural analysis demonstrated that the most effective strengthening mechanisms in the MMCs reinforced by graphene phenomena are a mismatch in the thermal expansion of the matrix and graphene and an Orowan looping mechanism.
- Despite the mechanical properties and microstructural analysis of MMCs reinforced by graphene that has been investigated intensively, their tribology is rarely considered and studied.
- The available investigations have shown that graphene can significantly decrease the wear rates and friction coefficients of MMCs with respect to the monolithic material.
- The improvement in the tribological features of MMCs, as well as the mechanical and thermophysical properties, strongly depend on the final graphene content.
- At higher graphene contents, graphene tends to form big agglomerates that leave some defects, like porosity, in the MMCs after fabrication, and accordingly deteriorate the final properties of MMCs.
- There is a critical limit for the final graphene content, until which the properties of MMCs can be improved, whereas at higher graphene contents, all the properties, such as thermal conductivity, electrical conductivity, mechanical properties and tribological behavior, are deteriorated. In addition to the graphene content, its other characteristics, such as the size of reinforcement, and spatial distribution, have shown to effect mechanical and tribological properties.
- It should be noted that these topics still bring a marked challenge to the materials scientists, and it would be worth mentioning that the production of metal matrix nanocomposites with a uniform distribution of graphene, strong interfacial bonding, without unfavorable reactions and with better isotropic properties is still a challenging topic.
- Each metallic material has some poor characteristics that limit its industrial applications. Theoretically and also on the lab-scale, it was found that through the addition of graphene, it would be possible to address those weakness and accordingly broad their application.
- Despite all the efforts that have been undertaken in the development of new MMNCs reinforced by graphene, the industrialization of these materials are faced with several challenges, and need more attempts.

## Figures and Tables

**Table 1:** Summary of research on metal matrix nanocomposites (MMNCs) reinforced by graphene.

Matrix	Reinforcement Content	Production Method	Features	Ref.
Al	0.3 wt.% graphene	BM and CPM	Tensile strength: 454 MPa Yield strengths: 322 MPa	[37]
	0.1 wt.% graphene	Blending, BM, HIP and extruding	Tensile strength: 270 MPa Yield strengths: 198 MPa	[38]
	0.3 wt.% GNSs	CPM and EX	Tensile strength: 249 MPa	[39]
	0.3 wt.% RGO	Compacting and HP	Elastic modulus: 90.1 GPa Hardness: 1.59 GPa	[40]
	2 wt.% GNSs	Liquid state	Tensile strength: 48.1 MPa Vickers hardness: 57.19 Elastic modulus: 87.93 GPa	[41]
	8–10 vol.% FLG	Electrochemical co-deposition	Reduction of resistivity of electrolytic Cu by 10–20%	[42]
	0.7 vol.% FLG	BM and HR	Tensile strength: 440 MPa	[43]
	1 wt.% FLG	BM, pre-compaction and hot compaction	Flexural stress: 750–800 MPa	[44]
	0.5–1.0 wt.% GNFs	Cryomilling and HEX	Tensile strength: 173–248 MPa	[45]
	0.25–1.0 wt.% GNPs	BM and CPM	Compressive strength: 180 MPa Vickers hardness: 70	[46]
	0.1–0.5 wt.% graphene	HP	Tensile strength: 95–110 MPa	[47]
	0.5, 1, 1.5, 2 wt.% graphene	Blending, cryo-milling, degassing and EX	Ultimate tensile strength: 248 MPa (1 wt.%) Yield strengths: 194 MPa Elongation: 8.3%	[48]
Al 2009	1 wt.% GNPs	CPM and multi-pass friction stir	Ultimate tensile strength: 514 MPa Yield strengths: 398 MPa	[49]
Al7055	1.0–5.0 wt.% graphene	SPS	Compressive strength: 600–1200 MPa Vickers hardness: 90–150	[50]
Cu	0.5, 1 vol.% graphene	BM and (HRDSR)	Yield strengths: 360.5 MPa (1 wt.%) Ultimate tensile strength: 425.5 MPa Elongation: 16.4%	[51]
	3, 5, 8, 12 vol.% graphene	Compacting and sintering	Yield strengths: 315 MPa Young’s modulus: 102 GPa	[52]
	0–4 vol.% GNPs	Molecular-level mixing process, SPS	Hardness: 1–1.8 GPa Electrical conductivity: 80–92% IACS Young’s modulus: 90–140 GPa	[53]
	1.3 wt.% GNPs	Electroless plating, SPS tensile	Strength: 485 MPa Elongation: 9% Young’s modulus: 104 GPa	[54]
	GNPs	Electrochemical deposition	Hardness: 111.2 HV Electrical conductivity: 89.2 % IACS	[55]
	0.5 wt.% GNPs	In-situ CVD	Tensile strength: 308 MPa	[56]
	0.3 wt.% RGO	GO fill in ‘brick-and-mortar’ Hot pressing	Yield strengths: 233 MPa Tensile strength: 218 MPa Young’s modulus: 109 GPa	[57]
Mg	0.3 wt.% graphene	Semi-powder metallurgy	Tensile strength: 208 MPa Failure strain: 10.9%	[58]
Mg–1 wt.% Al	0.3 wt.% GNPs	Powder metallurgy	Tensile strength: 246 MPa Yield strength: 178 MPa Hardness: 55 HV Young’s modulus: 13.84 GPa Elongation: 16.9%	[59]
	0.18 wt.% GNPs		Vickers hardness: 51 HV Young’s modulus: 12.18 GPa Yield strengths: 162 MPa Tensile strength: 223 MPa Failure strain: 15.2%	
	0.09 wt.% GNPs		Vickers hardness: 48 HV Young’s modulus: 13.40 GPa Yield strengths: 148 MPa Tensile strength: 206 MPa Failure strain: 10.5%	
Mg	1.0 vol.% GNPs	BM, SPS	Experimental density: 1.72 g/cm^3^ Hardness: 54 HV Compressive strength: 159 MPa	[60]
	2 vol.% GNPs		Experimental density: 1.74 g/cm^3^ Hardness: 63 HV Compressive strength: 201 MPa	
	5 vol.% GNPs		Experimental density: 1.75 g/cm^3^ Hardness: 50 HV Compressive strength: 123 MPa	
	1.2 vol.% GNPs	Liquid state ultrasonic and solid state friction stirring	Microhardness: 66 kg/mm^2^	[61]
	0.25, 0.75vol% GNPs	HP, HR	Tensile strength (0.25vol%): 160 MPa Tensile strength (0.75vol%): 179 MPa	[62]
Mg–1 wt.% Al	0.60 wt.% GNPs	Compaction, sintering and EX	Young’s modulus: 7.6 GPa Yield strength: 230 MPa Compress strength: 407 MPa Elongation: 13%	[63]
Mg–1 wt.% Al 1 wt.% Sn	0.18 wt.% GNPs	Semi-CPM and HEX	Tensile strength: 269 MPa Yield strength: 208 MPa	[58]
Mg–0.5 wt.% Al	0.18 wt.% GNPs	Semi powder metallurgy, HEX	Yield strengths: 173 MPa Tensile strength: 230 MPa Failure strain: 10.7% Vickers hardness: 55 HV	[64]
Mg—1.0 wt.% Al			Yield strengths: 190 MPa Tensile strength: 254 MPa Failure strain: 15.5% Vickers hardness: 58 HV	
Mg—1.5 wt.% Al			Yield strengths: 209 MPa Tensile strength: 268 MPa Failure strain: 12.7% Vickers hardness: 60 HV	
Mg alloy (ZK60)	0.05 wt.% GNPs	Facile melt stirring and HEX	Yield strength: 256 MPa	[65]
Mg—6Zn	0.5 wt.% GNPs	Disintegrated melt deposition	Yield strengths: 171 MPa Tensile strength: 295 MPa Fracture strain: 18% Ultimate compressive strength: 435 MPa	[66]
	1.5 wt.% GNPs		Yield strengths: 214 MPa Tensile strength: 313 MPa Fracture strain: 21% Compressive strength: 448 MPa	

FLG: Few-layer graphene, RGO: Reduced graphene, GNPs: Graphene nanoplatelets, GO: Graphene oxide, GNFs: Graphene nanoflakes, CPM: Conventional powder metallurgy, HC: Hot compaction, HR: Hot rolling, HEX: Hot extrusion, EX: Extrusion, BM: Ball-milling, HIP: Hot isostatic press, HP: Hot press, SPS: Spark plasma sintering, (HRDSR): High differential speed rolling, CVD: Chemical vapor deposition.

**Table 2:** The most important mechanical and physical characteristics of graphene.

Property	Graphene	Ref.
Resistivity	10^−6^ Ω·cm	[84]
Thermal conductivity	5.3 × 10^3^ W·m^−1^·K^−1^	
Transmittance	>95% for 2 nm thick film >70% for 10 nm thick film	[85]
Young’s modulus	0.5–1 TPa	
Thermal Expansion Coefficient	−6 × 10^−4^/K	
Young’s modulus	0.5–1 TPa	
Specific Surface area	2630 m^2^·g^−1^	
Ultimate tensile strength	130 GPa	
Thermal conductivity	5.3 × 10^3^ W·m^−1^·K^−1^	

**Table 3:** The features of common carbonaceous reinforcements [91,92,93,94,95].

Material	Thermal Conductivity (W·m^−1^·K^−1^)	Thermal Expansion Coefficient (10^6^ k^−1^)	Density (g·cm^−3^)	Melting Point (°C)	Vickers Hardness (HV)	Young’s Modulus (GPa)
Graphite	25–470	0.6–4.3	1.3–1.95	-	-	8–15
Diamond	2400	-	3.52	3550	8000	930
Graphene	5300	−0.8–0.7	1.8–2.2	-	-	1020
SWCNTs	Up to 2900	Negligible	1.8	-	-	1000

**Table 4:** Experimental mechanical features of carbonaceous nanomaterials.

Materials	Elastic Modulus, TPa	Tensile Strength, GPa	Experimental Methods	Ref.
Graphene	1.02	130	Nanoindentation in AFM	[102]
GNP	~1	~10–20	-	[104]
Arc-grown MWNT	1.8	-	Amplitudes of thermal vibrations of MWNTs placed inside TEM	[105]
SWNT	1.25	-	Amplitudes of thermal vibrations of SWNTs placed inside TEM	[106]
Arc-grown MWNT	1.28	-	Bending of pinned MWNT inside AFM	[107]
Arc-grown MWNT	0.81	-	Bending of pinned MWNT inside AFM	[108]
CVD-grown MWNT	0.027	-	Bending of pinned MWNT inside AFM	
Arc-grown MWNT	0.27–0.95	11–63	Tensile test of MWNT in SEM	[109]
SWNT rope	0.32–1.47	13–52	Tensile test of nanotube rope in SEM	[110]
Arc-grown MWNT	0.9	150	Tensile test of MWNT in TEM	[111]
Pyrolytic stripped CNF	0.18	2.90	Micro electromechanical device	[112]
Graphitized CNF	0.245	2.35	Micro electromechanical device	

**Table 5:** Reported characteristics of carbon fibre, carbon nanotube, carbon nanofiber and graphene [107,109,110,111,113,114,115].

Materials	Tensile Strength, GPa	Tensile Modulus, GPa	Thermal Conductivity, W·m^−1^·K^−1^
CF (T300; Cytec Thornel	3.65	231	8.5
SWNT	-	1000	3500
MWNT	150	270–950	500–2069
CNF (Graphitized)	2.35	245	1950
Graphene	130	1002	4840–5300

**Table 6:** Mechanical characteristics of graphite–copper and graphene nanosheets–copper nanocomposites [189].

Composites	Reinforcement (vol.%)	Microhardness (HV)	Bending Strength (MPa)	Relative Density (%)
Cu–GNPs	2.5	66.5	362.03	98.9
	5	69.2	294.39	98.5
	7.5	74.2	185.68	98.4
	10	68.9	149.01	98.2
Cu–GNSs	2.5	67.8	441.27	99.1
	5	71.7	301.16	98.9
	7.5	97.4	284.01	98.7
	10	56.8	211.85	97.5

**Table 7:** Mechanical properties of pure copper and its nanocomposites in the presence of various GNPs content [144].

Mechanical Features	Pure Cu	Cu-0.1 wt.% GNPs	Cu-0.2 wt.% GNPs	Cu-0.3 wt.% GNPs
Yield strength (MPa)	126	159	171	117
Tensile strength (MPa)	183	214	233	172
Elongation (%)	29	26	23	18
Vickers hardness (HV)	90	105	108.6	88

**Table 8:** A summary of the electrical conductivity of Cu–GNPs nanocomposites.

Content of Reinforcement	Electrical Conductivity (%IACS)	Production Technique	Ref.
1–5 wt.% MLG	78.5–61.5	Flake PM	[188]
0–4 vol.% GNPs	80–92	SPS	[53]
1 wt.% FLG	94 (at 600 °C) 81 (at 700 °C)	Mechanical milling and HP	[192]
2 vol.% GNPs	77	Sintering and HIPing	[193]
4 vol.% GNPs	72.5		
8 vol.% GNPs	67.5		

**Table 9:** A review of the characteristics of graphene-reinforced metal matrix nanocomposites.

Composite	Content of Reinforcement	Production Method	Properties	Ref.
Ni	0.5 wt.% 3D-GNs	In-situ high-temperature CVD, impregnation-reduction process and SPS	Relative density: 97.1% ± 0.1% Yield strengths: 344 ± 14 MPa Elongation: 35.4% ± 6.6%	[195]
	1.0 wt.% 3D-GNs		Relative density: 98.6% ± 0.2% Yield strengths: 474 ± 13 MPa Elongation: 25.5% ± 4.3%	
Ti	0.1 wt.% MLG	SPS and HR	Yield strengths: 857 ± 17 MPa Elongation: 19% ± 0.4% Ultimate tesile strength: 915 ± 15 MPa	[196]
	0.2 wt.% MLG		Yield strengths: 857 ± 17 MPa Elongation: 19% ± 0.4% Ultimate tensile strength: 915 ± 15 MPa	
Inconel 718	0.25 wt.% GNPs	Selective laser melting	Yield strengths: 912 MPa Elongation: 10.4% Tension strength: 1278 MPa Vickers hardness: 424 HV Wear rate: 8.505 (mm^3^·N^−1^·m^−1^))	[197]
	1.0 wt.% GNPs		Yield strengths: 1175 MPa Elongation: 4.3% Tensile strength: 1417 MPa Vickers hardness: 508 HV Wear rate: 8.505 (mm^3^·N^−1^·m^−1^)	
Ni_3_Al alloy	1.0 wt.% MLG	BM, CPM and SPS process	Relative higher hardness: 6.5 GPa Elastic modulus: 240 GPa	[198]
Ni	0.12 wt.% GO (1.2 nm)	Electro deposition	Thermal conductivity: 79 W m·K^−1^ Modulus: 252.76 GPa Hardness: 6.85 GPa	[200]
Fe	2 wt.% GO single layer	Laser sintering	Hardness: 580 kg·mm^−2^	[184]
ZK60	0.05wt.% GNPs	Melt stirring HEX	Yield strengths: 256 MPa (62% increase)	[65]
Ag–Cu–Ti alloy	GNSs	Melting	75% increase in the shear strength (of the graphite and Cu joint)	[199]
Steel	10 g/L GO	-	Wear volume: 2.5 × 10^−5^ mm^3^ (under water air) Wear volume: 7.8 × 10^−5^ mm^3^ (under nitrogen) Wear rate: 6.51 × 10^−8^ mm^3^/N.M (under water air) Wear rate: 2.08 × 10^−7^ mm^3^/N.M (under nitrogen) Coefficient of friction: 0.17 (under water air) Coefficient of friction: 0.16 (under nitrogen)	[201]
Ti	Ni–0.05 wt.% GNFs	BM, SPS, HR	Ultimate tensile strength: 793 ± 25 MPa Yield strengths: 748 ± 20 MPa Elongation: 18% ± 3%	[202]
	0.05 wt.% GNFs		Ultimate tensile strength: 722 ± 19 MPa Yield strengths: 651 ± 17 MPa Elongation: 19% ± 3%	
Ag	0.5 wt.% Ag-doped GNSs	Chemical reduction and CPM	Relative density: 94.87% Hardness: 76.10 HV Electrical conductivity: 98.62% (IACS)	[194]
	Ag-doped GNSs (1.5 wt.%)		Relative density: 94.80% Hardness: 57.35 HV Electrical conductivity: 94.83% (IACS)	
	Ag-doped GNSs (3 wt.%)		Relative density: 92.30% Hardness: 65.42 HV Electrical conductivity: 92.80% (IACS)	
Sn–2.5Ag–0.7Cu	Ni–0.03–0.05wt.% GNSs	MA	Optimum strength-toughness Optimum wettability Electrical conductivity: 15.78–15.46% IACS Increase of ultimate tension stress	[203]

Three-dimensional graphene networks (3D GNs); graphene nanoplates (GNPs); multilayer graphene (MLG); graphene oxide (GO); graphene nanoflakes (GNFs); graphene nanosheets (GNSs); hot-rolling (HR); spark plasma sintering (SPS); mechanical alloying (MA); conventional powder metallurgy (CPM), ball milling (BM); chemical vapor deposition (CVD).

**Table 10:** An overview of the potential application of MMNCs reinforced by graphene.

MMNCs	Properties and Applications	Ref.
Cu/Graphene	High thermal and electrical conductive foil for electronic packaging	[205]
Mg/graphene	Ultra-high performance parts for automotive industries	[206]
Au/Graphene	H_2_O_2_ biosensor	[207]
Si/Graphene	Anode materials for Li-ion battery	[208]
Pt/Graphene	Super capacitor-fuel cell	[209]
